# Impact of diabetes on emergency care of acute myocardial infarction patients during the coronavirus disease 2019 pandemic: a nationwide population-based study

**DOI:** 10.3389/fpubh.2023.1151506

**Published:** 2023-04-26

**Authors:** Eyun Song, Jeongeun Hwang, Sung Joon Park, Min Jeong Park, Ahreum Jang, Kyung Mook Choi, Sei Hyun Baik, Hye Jin Yoo

**Affiliations:** ^1^Division of Endocrinology and Metabolism, Department of Internal Medicine, Korea University College of Medicine, Seoul, Republic of Korea; ^2^Division of Medical Oncology, Department of Internal Medicine, Korea University College of Medicine, Seoul, Republic of Korea; ^3^Department of Biomedical Research Center, Korea University Guro Hospital, Seoul, Republic of Korea; ^4^Department of Emergency Medicine, Korea University College of Medicine, Seoul, Republic of Korea

**Keywords:** diabetes, acute myocardial infarction, emergency department, COVID-19, pandemic

## Abstract

**Background:**

Although acute myocardial infarction (AMI) requires timely intervention, limited nationwide data is available regarding the association between disruption of emergency services and outcomes of patients with AMI during the coronavirus disease 2019 (COVID-19) pandemic. Moreover, whether diabetes mellitus (DM) adversely affects disease severity in these patients has not yet been investigated.

**Methods:**

This nationwide population-based study analyzed 45,648 patients with AMI, using data from the national registry of emergency departments (ED) in Korea. Frequency of ED visits and disease severity were compared between the COVID-19 outbreak period (year 2020) and the control period (the previous year 2019).

**Results:**

The number of ED visits by patients with AMI decreased during the first, second, and third waves of the outbreak period compared to the corresponding time period in the control period (all *p*-values < 0.05). A longer duration from symptom onset to ED visit (*p* = 0.001) and ED stay (*p* = 0.001) and higher rates of resuscitation, ventilation care, and extracorporeal membrane oxygen insertion were observed during the outbreak period than during the control period (all *p*-values < 0.05). These findings were exacerbated in patients with comorbid DM; Compared to patients without DM, patients with DM demonstrated delayed ED visits, longer ED stays, more intensive care unit admissions (*p* < 0.001), longer hospitalizations (*p* < 0.001), and higher rates of resuscitation, intubation, and hemodialysis (all *p*-values < 0.05) during the outbreak period. While in-hospital mortality was similar in AMI patients with and without comorbid DM during the two periods (4.3 vs. 4.4%; *p* = 0.671), patients with DM who had other comorbidities such as chronic kidney disease or heart failure or were aged ≥ 80 years had higher in-hospital mortality compared with those without any of the comorbidities (3.1 vs. 6.0%; *p* < 0.001).

**Conclusion:**

During the pandemic, the number of patients with AMI presenting to the ED decreased compared with that of the previous year, while the disease severity increased, particularly in patients with comorbid DM.

## 1. Introduction

Since the first case of the coronavirus disease 2019 (COVID-19), caused by the severe acute respiratory coronavirus 2 (SARS-CoV-2), was reported in December 2019 in China (1), COVID-19 has cripplingly and rapidly spread across the entire world. The World Health Organization (WHO) declared COVID-19 a global pandemic on March 11, 2020 (2), and many countries imposed rigorous measures to restrict its spread. In Korea, facilities with confirmed cases were temporarily closed, including the emergency department (ED) (3, 4). In addition, healthcare resources were primarily assigned to managing patients with COVID-19-related diseases, limiting the timely and adequate treatment of other critically ill patients (5–7).

Acute myocardial infarction (AMI) is a sequelae of ischemic cascade and myocardial necrosis that requires prompt revascularization (8). Several reports have described a significant decline in hospitalization or percutaneous coronary intervention (PCI) procedures in patients with AMI during the COVID-19 pandemic (9–13). Although there are studies assessing this phenomenon with a focus on emergency care services (14–17), most are local or regional level analyses. Few studies have analyzed nationwide data, covering baseline patient characteristics and clinical outcomes of patients with AMI. Moreover, there is a paucity of reports on how diabetes mellitus (DM) affects the outcomes of patients with AMI presenting to the ED during the COVID-19 pandemic. Given the considerable increase in disease severity and mortality of COVID-19 in patients with DM (18), we hypothesized that it would be valuable to evaluate whether DM has an impact on ED visits, patient care, and patient outcomes of AMI during the COVID-19 outbreak. Thus, this study aimed to determine the patterns and changes in the utilization of ED in patients with AMI during the pandemic and to particularly examine whether the outcomes of these patients were affected by comorbid DM, using the nationwide ED registry data in Korea.

## 2. Materials and methods

### 2.1. Data source

This nationwide population-based cohort study used data from the National Emergency Department Information System (NEDIS) registry of the National Emergency Medical Center (NEMC) in South Korea. Since 2016, demographic and clinical data of patients visiting all types of emergency healthcare facilities—regional emergency medical centers, local emergency medical centers, and local emergency medical institutions—have been prospectively collected in NEDIS (19). This study used data collected from 2019 to 2020; 401/402 (99.8%) and 403/403 (100%) emergency healthcare facilities participated in the NEDIS registry in 2019 and 2020, respectively. Approximately 68 items are inputted in the NEDIS registry, including age (as a categorical variable by 5-year interval), sex, address, insurance type, time and date of visit, chief complaint, time and date of symptom onset, vital signs at presentation, initial and final level of severity [as per the Korean Triage and Acuity Scale (KTAS), which is used to determine emergency patient priority], International Classification of Diseases Tenth Revision (ICD-10) based diagnosis, time of ED discharge, distribution after emergency care, procedures performed during admission, result of admission (discharge, transfer, or expired), and time and date of discharge. The accuracy of the collected data is monitored annually by the NEMC (19).

### 2.2. Study design

**Figure 1:**
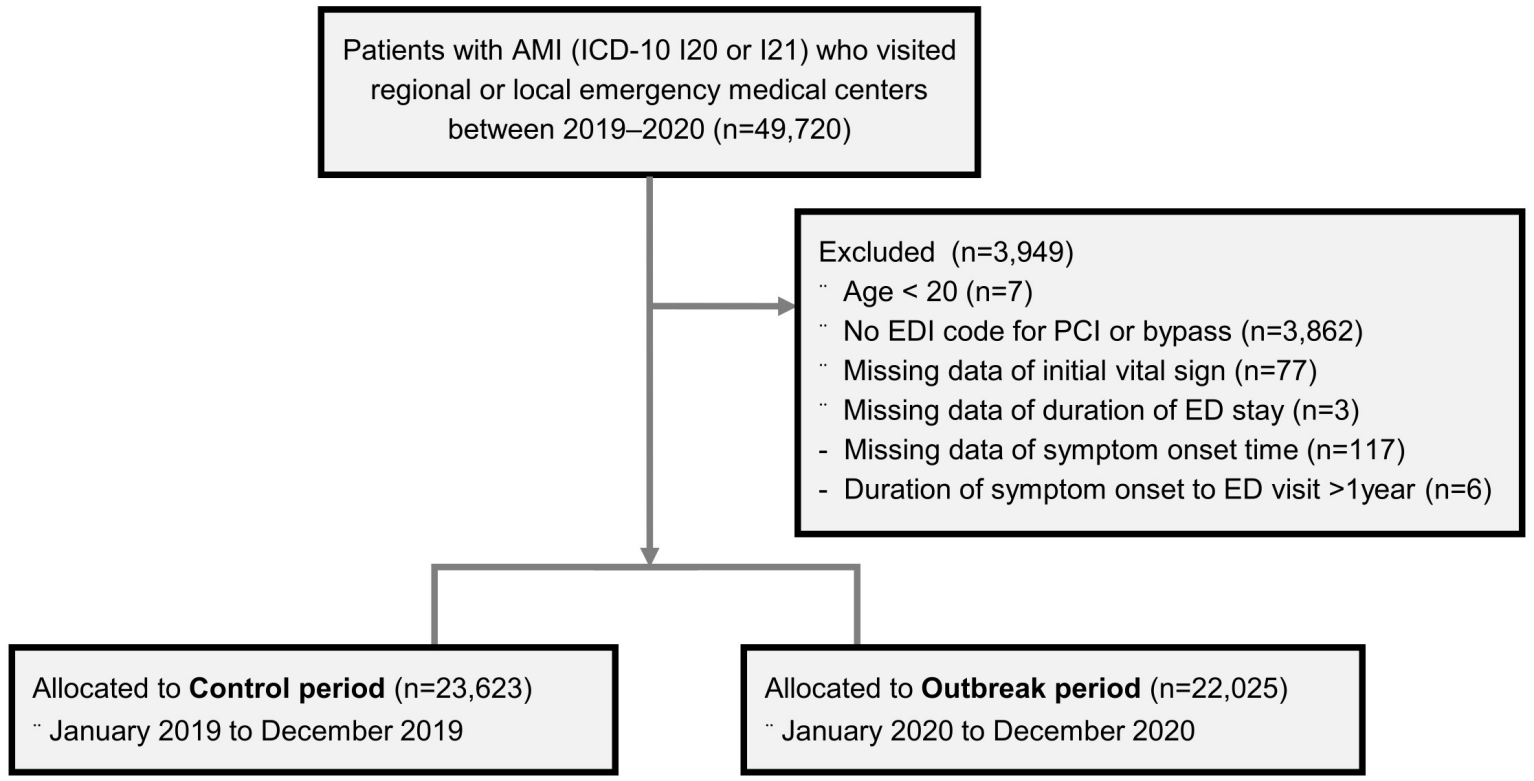
Study population.

Patients who visited the regional or local emergency medical centers between 2019 and 2020 and were given a primary diagnosis of ICD-10 codes I20 or I21 at discharge from the ED or hospital were initially screened (*n* = 49,720; Figure 1). To improve the accuracy of identifying patients with AMI, only those with procedure codes for percutaneous coronary intervention (PCI) or artery bypass surgery were selected (Supplementary Table 1). The following patients were excluded from the study: patients aged < 20 years; those with missing data on initial vital signs, time of ED discharge, and symptom onset time; and those with a remote symptom onset time of > 1 year. Because the first COVID-19 case was confirmed on January 20, 2020, in Korea, the period between January and December 2020 was defined as the “outbreak period,” and the prior year, i.e., the period between January and December 2019 was deemed the “control period” to provide a reference for comparison. A total of 45,648 patients were eligible for analysis and allocated either to the “control period” (*n* = 23,623) or the “outbreak period” (*n* = 22,025), according to the date of their ED visit. The outbreak period was further divided into four time periods according to the number of confirmed COVID-19 cases: the first wave, weeks 7–16; the post-first wave, weeks 17–30; the second wave, weeks 31–42, and the third wave, weeks 43–53 (until the last week of data availability, although the third wave continued to the year 2021). The study protocol was approved by the Institutional Review Board of Korea University (IRB number 2022GR0171).

### 2.3. Study outcomes and definitions

Disease severity of patients who visited the ED with AMI was assessed based on the following factors: final KTAS score at ED discharge, major procedures performed [intubation, hemodialysis, and extracorporeal membrane oxygenation (ECMO)], and in-hospital mortality. KTAS ranges from level 1 to 5, with level 1 being the most severe: level 1, resuscitation; level 2, emergency; level 3, urgency; level 4, less urgency; and level 5, non-urgency. The procedure codes for intubation, hemodialysis, and ECMO are listed in Supplementary Table 2. Study outcomes were compared between the control and outbreak periods and between patients with and without DM. DM was defined as a primary or secondary diagnosis of ICD-10 codes E10, E11, E12, E13, or E14. In addition, patients with other severe comorbidities such as chronic kidney disease (CKD) stage 4 or 5 (ICD-10 codes N18.4 or N18.5), heart failure (ICD-10 code I50), or old age (≥ 80 years) were compared with those without any of the comorbidities.

### 2.4. Statistical analysis

R statistics software version 4.1.3 (R Foundation for Statistical Computing, Vienna, Austria) and R packages, including ggplot2, lubridate, readxl, and dplyr, were used for statistical analysis. Two-sample tests for equality of proportion were performed to compare the rates, including age groups, sex, KTAS level, distribution after emergency care, PCI, bypass surgery, intubation, hemodialysis, ECMO usage, and in-hospital mortality rates. Welch’s two-sample *t*-tests were performed to compare numerical variables, including blood pressure, pulse rate, respiratory rate, and body temperature. Mann–Whitney *U*-test with continuity correction was performed to compare continuous variables, including hours taken from symptom onset to ED visit, duration of ED stay, and duration of hospital stay.

## 3. Results

### 3.1. Frequency of ED visits of patients with AMI

**Figure 2:**
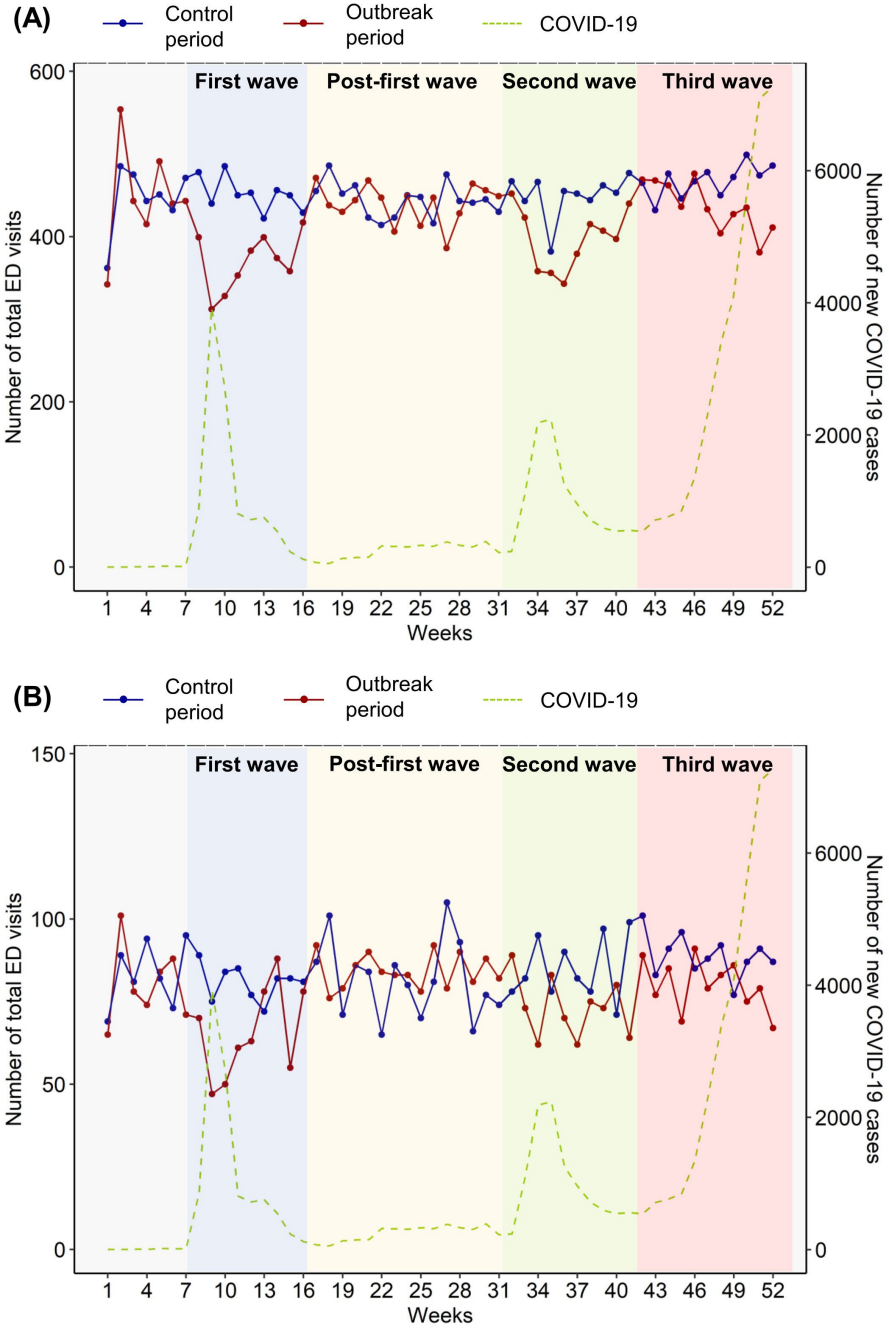
Frequency of emergency department (ED) visits **(A)** among all patients with acute myocardial infarction (AMI) during the outbreak (January 2020–December 2020) and control (January 2019–December 2019) periods and **(B)** among patients with AMI and underlying DM during the outbreak period. The blue line represents the weekly number of total ED visits during the control period, while the red line represents that of the same weeks during the outbreak period. The green dashed line indicates the total number of new COVID-19 cases in South Korea, regardless of ED visits or AMI or DM status. The blue background denotes the first wave of the COVID-19 outbreak in South Korea, while the green and pink backgrounds represent the second and third waves, respectively. ED, emergency department; AMI, acute myocardial infection; DM, diabetes mellitus.

According to the NEDIS registry, there were a total of 6,146,688 ED visits in 2019 and 4,809,661 in 2020. During the outbreak period, 22,025 patients visited the ED owing to AMI and underwent PCI or bypass surgery, while 23,623 patients visited during the control period. The frequency of ED visits during the outbreak and control periods is shown in Figure 2A. There was a decrease in ED visits of patients with AMI during the first, second, and third waves of the outbreak period, while relatively stable visits were observed throughout the control period. When comparing the mean number of ED visits between each of the three waves of the outbreak period and a corresponding time period in the control period, significantly lesser visits were observed during the first, second, and third waves in the outbreak period compared to the corresponding control periods as follows: 451 (control) vs. 369 (first wave) visits (*p* < 0.001), 450 (control) vs. 399 (second wave) visits (*p* = 0.004), and 468 (control) vs. 433 (third wave) visits (*p* = 0.008). However, no difference was observed during the post-first wave (446 visits in the control period vs. 446 in the outbreak period; *p* = 0.516). Similar findings were noted in patients with underlying DM (Figure 2B), with drops in ED visits during the three waves of the outbreak period with the corresponding control periods: 81 (control) vs. 66 (first wave) visits (*p* = 0.012), 87 (control) vs. 73 (second wave) visits (*p* = 0.004), and 88 (control) vs. 79 (third wave) visits (*p* = 0.009). However, for these patients, comparable numbers of ED visits were observed during the post-first wave (82 in the control period vs. 84 in the outbreak period, *p* = 0.353).

### 3.2. Comparison between the outbreak and control periods

The demographics and baseline clinical characteristics of patients with AMI according to their ED visit periods are summarized in Table 1. The distribution of patients in the age groups was similar between the two periods, with no significant differences between the sexes (*p* = 0.100). Almost all patients underwent PCI (99.8% in the control period and 99.9% in the outbreak period; *p* = 0.471). The time from symptom onset to ED visit was significantly longer in the outbreak period than in the control period (4.20 vs. 4.37 h, respectively, *p* = 0.001). In addition, the duration of ED stay was significantly longer during the outbreak period than during the control period (3.60 vs. 3.80 h, respectively, *p* = 0.001). After emergency care, patients were either transferred due to medical shortage, admitted to the general ward, admitted to the intensive care unit (ICU), or died; there was no difference in these outcomes between the two periods. The duration of total hospital stay was also similar between the outbreak and control periods (3.60 vs. 3.67 days, respectively, *p* = 0.058).

**Table 1:** Baseline clinical characteristics of patients with AMI during the control and the outbreak periods.

Characteristics	Control period (*n* = 23,623)	Outbreak period (*n* = 22,025)	*p-*value
Age (years)			
20–29	44 [0.2%]	52 [0.2%]	0.245
30–39	444 [1.9%]	410 [1.9%]	0.887
40–49	2,288 [9.7%]	1,998 [9.1%]	0.025
50–59	5,268 [22.3%]	4,799 [21.8%]	0.188
60–69	6,336 [26.8%]	6,140 [27.9%]	0.011
70–79	5,745 [24.3%]	5,232 [23.8%]	0.158
80–89	3,222 [13.6%]	3,117 [14.2%]	0.113
90	276 [1.2%]	277 [1.3%]	0.383
Sex			0.100
Male [*n* (%)]	17,049 [72.2%]	16,047 [72.9%]	
Female [*n* (%)]	6,574 [27.8%]	5,978 [27.1%]	
Procedure			
PCI [*n* (%)]	23,587 [99.8%]	21,997 [99.9%]	0.471
Bypass surgery [*n* (%)]	70 [0.30%]	78 [0.35%]	0.277
Vital sign			
Systolic BP (mmHg) [mean (SD)]	136 (± 33)	135 (± 37)	0.009
Diastolic BP (mmHg) [mean (SD)]	80 (± 20)	80 (± 22)	0.078
Pulse rate (beats/min) [mean (SD)]	80.1 (± 21.7)	79.6 (± 23.2)	0.022
Respiratory rate (beats/min) [mean (SD)]	19.5 (± 4.0)	19.1 (± 4.3)	< 0.001
Temperature (°C) [mean (SD)]	36.1 (± 3.5)	36.1 (± 3.6)	0.328
Symptom onset to ED visit (hours) [median (IQR)]	4.20 (1.18–24.0)	4.37 (1.35–24.0)	0.001
Duration of ED stay (hours) [median (IQR)]	3.60 (1.70–7.15)	3.80 (1.78–8.02)	< 0.001
Distribution after emergency care (%)			
Death	55 [0.23%]	50 [0.23%]	0.897
Transfer d/t medical resource shortage	7 [0.03%]	5 [0.02%]	0.648
Admission to GW	9,810 [41.5%]	9,036 [41.0%]	0.277
Admission to ICU [*n* (%)]	13,485 [57.1%]	12,713 [57.7%]	0.169
Hospital stays (days) [median (IQR)]	3.67 (2.07–5.88)	3.60 (2.02–5.83)	0.0578

AMI, acute myocardian infection; PCI, percutaneous coronary intervention; ICU, intensive care unit; BP, blood pressure, SD, standard deviation; IQR, interquartile range; ED, emergency department; GW, general ward.

**Figure 3:**
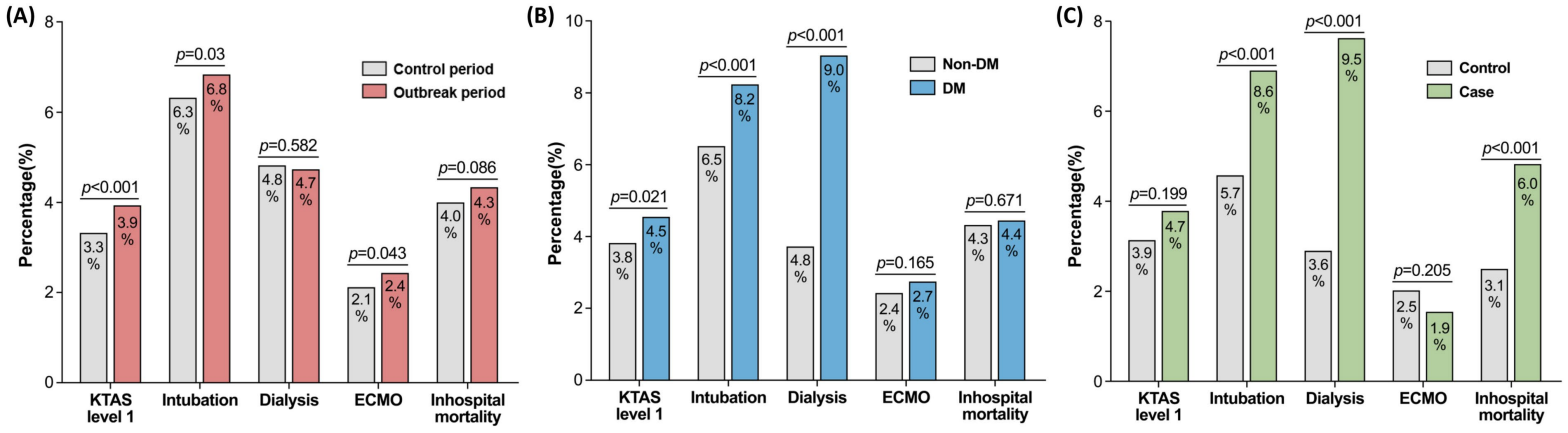
Differences in clinical outcomes in patients with AMI **(A)** between the outbreak and control periods and **(B)** according to the presence/absence of comorbid DM. **(C)** shows the comparison between patients with DM and either CKD, heart failure, or age ≥ 80 years (case) and those without DM, CKD, heart failure and age < 80 years (control). Two-sample tests for equality of proportion were performed to compare the rates. AMI, acute myocardial infection; DM, diabetes mellitus.

Differences in major clinical outcomes were compared in patients with AMI between the two periods, as shown in Figure 3A. The proportion of patients with KTAS level 1 was significantly higher among those who visited the ED during the outbreak period than among those who visited during the control period (3.3 vs. 3.9%, respectively, *p* < 0.001). Moreover, the intubation (6.3 vs. 6.8%, respectively, *p* = 0.03) and ECMO insertion rates (2.1 vs. 2.4%, respectively, *p* = 0.043) were higher in the outbreak period than that in the control period. The in-hospital mortality was 4.3% in the outbreak and 4.0% in the control periods, although the difference was not statistically significant (*p* = 0.086).

### 3.3. Clinical differences according to DM status during the outbreak period

Given the increased severity of the disease course in patients with AMI during the outbreak period, we further assessed the clinical differences according to their DM status. In 2020, 4,071 patients with DM and 17,954 without DM visited the ED for AMI (Table 2). The proportion of older patients and women was higher in the DM group than in the non-DM group (*p* = 0.002). The time from symptom onset to ED arrival (4.25 vs. 4.90 h; *p* < 0.001) and duration of ED stay (3.78 vs. 4.05 h; *p* = 0.002) was significantly longer in patients with DM than in those without DM. Notably, compared to patients without DM, more patients with DM were admitted to the ICU (57.1 vs. 60.3%, respectively, *p* < 0.001) and were less frequently admitted to the general ward (41.4 vs. 39.3%, respectively, *p* = 0.012). In addition, the duration of hospital stay was significantly longer in the DM group than in the non-DM group (3.46 vs. 4.01 days, respectively *p* < 0.001).

**Table 2:** Baseline characteristics of patients with AMI with and without comorbid DM during the outbreak period.

Characteristics	Non-DM (*n* = 17,954)	DM (*n* = 4,071)	*p-*value
Age (years)			
20–29	49 [0.3%]	3 [0.1%]	0.018
30–39	368 [2.0%]	42 [1.0%]	< 0.001
40–49	1,716 [9.6%]	282 [6.9%]	< 0.001
50–59	3,975 [22.1%]	824 [20.2%]	0.008
60–69	4,942 [27.5%]	1,198 [29.4%]	0.014
70–79	4,117 [22.9%]	1,115 [27.4%]	< 0.001
80–89	2,537 [14.1%]	580 [14.2%]	0.847
90	250 [1.4%]	27 [0.7%]	< 0.001
Sex			0.002
Male [*n* (%)]	13,159 [73.3%]	2,888 [70.9%]	
Female [*n* (%)]	4,795 [26.7%]	1,183 [29.1%]	
Procedure			
PCI [*n* (%)]	17,932 [99.9%]	4,065 [99.9%]	0.688
Bypass surgery [*n* (%)]	58 [0.32%]	20 [0.49%]	0.103
Vital sign			
Systolic BP (mmHg) [mean (SD)]	136 (± 37)	135 (± 38)	0.601
Diastolic BP (mmHg) [mean (SD)]	80 (± 22)	79 (± 23)	0.007
Pulse rate (beats/min) [mean (SD)]	79 (± 23)	82 (± 24)	< 0.001
Respiratory rate (beats/min) [mean (SD)]	19.1 (± 4.2)	19.5 (± 4.3)	< 0.001
Temperature (°C) [mean (SD)]	36.1 (± 3.6)	36.1 (± 3.5)	0.865
Symptom onset to ED visit (hours) [median (IQR)]	4.25 (1.31–24.0)	4.90 (1.48–25.9)	< 0.001
Duration of ED stay (hours) [median (IQR)]	3.78 (1.75–7.95)	4.05 (1.93–8.33)	0.002
Distribution after emergency care (%)			
Death	44 [0.25%]	6 [0.15%]	0.237
Transfer d/t medical resource shortage	14 [0.08%]	0 [0%]	–
Admission to GW	7,437 [41.4%]	1,599 [39.3%]	0.012
Admission to ICU [*n* (%)]	10,260 [57.1%]	2,453 [60.3%]	< 0.001
Hospital stays (days) [median (IQR)]	3.46 (1.95–5.66)	4.01 (2.68–6.93)	< 0.001

AMI, acute myocardian infection; PCI, percutaneous coronary intervention; ICU, intensive care unit; BP, blood pressure, SD, standard deviation; IQR, interquartile range; ED, emergency department; GW, general ward; DM, diabetes mellitus.

When comparing the major clinical outcomes based on DM status (Figure 3B), the proportion of patients with KTAS level 1 was 4.5% in the DM group and 3.8% in the non-DM group (*p* = 0.021). The intubation rate was significantly higher (6.5 vs. 8.2%, respectively, *p* < 0.001) and more patients underwent dialysis (3.7 vs. 9.0%, respectively, *p* < 0.001) in the DM group compared to the non-DM group. Despite this, the in-hospital mortality was similar between the two groups (4.3 vs. 4.4%; *p* = 0.671).

### 3.4. Major outcomes in patients with combined comorbidities during the outbreak period

In adjunction with DM, patients with other severe comorbidities including CKD stage 4 or 5, heart failure, or old age were compared with those without any of them (Table 3). The time from symptom onset to ED arrival were significantly longer in patients with combined comorbidities than that of the controls (3.92 vs. 5.52 h; *p* < 0.001) as well as the duration of ED stay (3.65 vs. 4.15 h; *p* < 0.001). Admission rate to ICU were also higher in patients with combined comorbidities (57.8 vs. 62.7%; *p* = 0.003). Major clinical outcomes were compared between the two groups (Figure 3C) which showed higher rates of intubation (5.7 vs. 8.6%; *p* < 0.001) and dialysis (3.6 vs. 9.5%; *p* < 0.001) in patients with severe comorbidities than the controls. Accordingly, the in-hospital mortality was significantly higher in patients with combined comorbidities than those with none (3.1 vs. 6.0%; *p* < 0.001).

**Table 3:** Baseline characteristics in patients with and without combined comorbidities during the outbreak period.

Characteristics	Control (*n* = 13,430)	Cases (*n* = 970)	*p-*value
Age (years)			
20–29	40 [0.3%]	0 [0.0%]	0.089
30–39	330 [2.5%]	3 [0.3%]	< 0.001
40–49	1,524 [11.3%]	39 [4.0%]	< 0.001
50–59	3,478 [25.9%]	92 [9.5%]	< 0.001
60–69	4,394 [32.7%]	120 [12.4%]	< 0.001
70–79	3,664 [27.3%]	112 [11.5%]	< 0.001
80–89	0 [0.0%]	577 [59.5%]	< 0.001
90	0 [0.0%]	27 [2.8%]	< 0.001
Sex			< 0.001
Male [*n* (%)]	10,459 [77.9%]	534 [55.1%]	
Female [*n* (%)]	2,971 [22.1%]	436 [44.9%]	
Procedure			
PCI [*n* (%)]	13,416 [99.9%]	969 [99.9%]	0.991
Bypass surgery [*n* (%)]	45 [0.34%]	3 [0.31%]	0.893
Vital sign			
Systolic BP (mmHg) [mean (SD)]	136 (± 37)	139 (± 31)	0.253
Diastolic BP (mmHg) [mean (SD)]	81 (± 22)	80 (± 18)	< 0.001
Pulse rate (beats/min) [mean (SD)]	79 (± 22)	84 (± 22)	< 0.001
Respiratory rate (beats/min) [mean (SD)]	18.9 (± 4.1)	20.2 (± 3.3)	< 0.001
Temperature (°C) [mean (SD)]	36.1 (± 3.6)	36.5 (± 0.6)	0.057
Symptom onset to ED visit (hours) [median (IQR)]	3.92 (1.20–23.1)	5.52 (1.87–26.2)	< 0.001
Duration of ED stay (hours) [median (IQR)]	3.65 (1.65–7.73)	4.15 (2.02–8.18)	0.001
Distribution after emergency care (%)			
Death	0 [0.0%]	0 [0.0%]	–
Transfer d/t medical resource shortage	0 [0.0%]	0 [0.0%]	–
Admission to GW	5,663 [42.2%]	362 [37.3%]	0.003
Admission to ICU [*n* (%)]	7,766 [57.8%]	608 [62.7%]	0.003
Hospital stays (days) [median (IQR)]	3.17 (1.90–5.18)	4.68 (2.82–8.38)	< 0.001

Cases are patients with DM and either CKD, heart failure, or age ≥ 80 years and controls are subjects without DM, CKD, heart failure and age < 80 years.

DM, diabetes mellitus; CKD, chronic kidney disease; PCI, percutaneous coronary intervention; ICU, intensive care unit; BP, blood pressure, SD, standard deviation; IQR, interquartile range; ED, emergency department; GW, general ward; DM, diabetes mellitus.

## 4. Discussion

This study analyzed nationwide data on emergency care facilities in Korea to evaluate the patterns of ED visits and clinical outcomes of patients with AMI visiting the ED, with a particular focus on the impact of comorbid DM in patients with AMI, during the COVID-19 pandemic. We observed a decrease in patients with AMI presenting to the ED during the outbreak period compared with the previous year. Moreover, significantly longer durations of both symptom onset to ED visit and ED stay were noted, in addition to higher rates of resuscitation, ventilation care, and ECMO insertion, during the outbreak period compared to the control period. These findings were present and exacerbated in patients with AMI and underlying DM; compared to patients without DM, patients with AMI and underlying DM were more frequently admitted to the ICUs, hospitalized longer, and had higher rates of resuscitation, intubation, and hemodialysis during the pandemic. Notably, when patients with DM had other comorbidities such as CKD or heart failure or were aged ≥ 80 years, their in-hospital mortality was increased compared to those without any of the comorbidities. Taken together, while there were fewer patients with AMI who presented to the ED during the COVID-19 outbreak, their disease severity was higher, especially in patients with AMI and underlying DM. To the best of our knowledge, this is the largest study of its kind to date that used a comprehensive national dataset to cover various aspects of the utilization of emergency care services as well as clinical outcomes of patients with AMI, while highlighting the impact of underlying DM in patients with AMI, during the COVID-19 outbreak.

Unique trends in ED visits have been reported from previous respiratory infection outbreaks. During the SARS outbreak in 2003, a reduction in total ED visits was observed (20, 21), although respiratory illness-related visits increased in adults and teenagers (20). However, during the 2009 influenza A pandemic, a marked surge in the number of ED visits was observed (22, 23). During the Middle East respiratory syndrome–coronavirus (MERS–CoV) epidemic in Korea in 2015, the number of non-urgent ED visits decreased (24, 25), whereas no change was detected in visits due to severe diseases (25). During the COVID-19 pandemic, substantial decreases in the volume of ED visits has been reported worldwide (26–29), and this phenomenon was similarly observed in a subgroup of patients with AMI (11, 14, 17, 30). Likewise, our results showed fewer ED visits in patients with AMI, and notably, there was a marked decrease during the first wave of the outbreak. This is in line with previous reports that showed decreased visits during the first week of the pandemic (30, 31). Therefore, we postulate that the patients must have particularly feared contracting an in-hospital infection with this strange and unfamiliar virus. Furthermore, the shortage and lockdown of emergency facilities may partially explain the considerably reduced number of visits during the first wave of the pandemic.

Our study showed that during the outbreak period, the volume of ED visits by patients with AMI decreased, while the severity of the disease increased. Disease severity was assessed with the final KTAS scores at ED discharge; the proportion of major procedures performed, such as intubation, hemodialysis, and ECMO; and in-hospital mortality. KTAS scores and the proportion of major procedures performed were higher during the outbreak than during the control period. Increased rates of intubation and ECMO insertion in patients with AMI who underwent revascularization may reflect higher rates of cardiac arrest, as previously reported (32–34). These increases in disease severity may be due to situation-related or direct infection-related factors during the pandemic. Restricted access to the emergency medical system and patients’ hesitation to visit the ED due to COVID-19 may have delayed the arrival time to the ED, as observed in our study and other previous studies (16, 30). Thus, relatively high-risk patients could have presented to the ED during the outbreak period. Besides the delay of ED visits, delays in time to revascularization and a lower proportion of procedures performed during the outbreak have been reported (30, 35), which may have collectively led to increased disease severity. Unfortunately, time-to-procedure data are not available in the current NEDIS dataset. Concerning the direct infection-related factors, studies demonstrate high rates of myocardial injury in patients confirmed with COVID-19 (36, 37), and the possible underlying mechanisms are type 1 myocardial infarction or myocarditis enhanced by viral infection (38–40) and type 2 myocardial infarction triggered by increased oxygen demand due to an inflammatory response (41). However, whether these factors played a role in our findings could not be confirmed, as no information was given regarding the patients’ COVID-19 status in the database.

Another important finding of this study is that DM exacerbated the clinical course of patients with AMI during the pandemic, which has not yet been evaluated in other studies. It is well established that DM is a common poor prognostic factor in COVID-19 cases (42–44). As expected, patients with comorbid DM and AMI had a higher disease severity during the pandemic (higher rates of ICU admission, resuscitation, ventilation care, and hemodialysis) than that of those with AMI but no DM. Various barriers to accessing medical services due to COVID-19 restrict patients with DM visits to hospitals for routine follow-up and access to their prescriptions, which generally hampers their regular diabetes care (45, 46). Complete care processes are crucial in patients with DM to lower complication rates and improve survival (47–49); consequently, disruption of routine medical care practices during the pandemic may have adversely affected the control of major risk factors such as blood pressure, weight, and lipid panels (46), thus worsening the severity of complications, including cardiovascular disease. Although the in-hospital mortality was comparable between patients with and without DM, it is noteworthy that when patients with DM had other severe comorbidities such as CKD, heart failure, or age ≥ 80 years, the in-hospital mortality was significantly increased compared with those without any of the comorbidities. Particular attention should be paid on these patients with combined severe comorbidities.

This study has some limitations. First, claims data from the NEDIS registry was used, which limits the assessment of laboratory results or medication use. Second, to improve the accuracy of the diagnosis of AMI, we selected patients with both relevant ICD-10 codes and procedure codes; thus, we could not compare the proportion of PCIs or bypass surgeries performed on patients with AMI during the outbreak and control periods. Despite these limitations, the strength of our study lies in comprehensively addressing all aspects of emergency care of patients with AMI during the COVID-19 period—details of changes in pattern in the frequency of ED visits were calculated at 1-week intervals and compared with the corresponding control periods. Additionally, various clinical characteristics of the patients with AMI, including the procedures that have been performed, with a focus on the impact of comorbid DM, were elucidated, using a nationwide dataset of emergency care facilities.

## 5. Conclusion

In conclusion, during the 2020 COVID-19 outbreak, the number of patients with AMI presenting to the ED decreased compared with that of the previous year, while the disease severity increased, particularly in patients with comorbid DM. This study sheds light on the fact that careful attention should be paid beyond the COVID-19 infection itself and to the non-COVID-19-related diseases such as AMI, especially when high-risk co-morbidity exists, such as DM.

## Data availability statement

Publicly available datasets were analyzed in this study. This data can be found at: National Emergency Department Information System repository in South Korea (http://dw.nemc.or.kr).

## Ethics statement

The studies involving human participants were reviewed and approved by Institutional Review Board of Korea University (IRB number 2022GR0171). Written informed consent for participation was not required for this study in accordance with the national legislation and the institutional requirements.

## Author contributions

ES researched the data, wrote, reviewed and edited the manuscript. JH researched and analyzed the data, and reviewed the manuscript. SP researched the data and contributed to methodology. MP, AJ, KC, and SB reviewed and edited the manuscript. HY researched the data, and reviewed and edited the manuscript. All authors contributed to the article and approved the submitted version.

## Funding

This research was supported by the Basic Science Research Program through the National Research Foundation of Korea (NRF), South Korea, funded by the Ministry of Education (NRF-2020R1I1A1A01072592 and NRF-2021R1A2C2008792) and by the Korea Medical Device Development Fund grant funded by the Korean government (the Ministry of Science and ICT, the Ministry of Trade, Industry and Energy, the Ministry of Health and Welfare, and the Ministry of Food and Drug Safety; Project Number: 9991007469, KMDF_PR_20200901_0233).

## Conflict of interest

The authors declare that the research was conducted in the absence of any commercial or financial relationships that could be construed as a potential conflict of interest.

## Publisher’s note

All claims expressed in this article are solely those of the authors and do not necessarily represent those of their affiliated organizations, or those of the publisher, the editors and the reviewers. Any product that may be evaluated in this article, or claim that may be made by its manufacturer, is not guaranteed or endorsed by the publisher.
